# Application of Bayesian genomic prediction methods to genome-wide association analyses

**DOI:** 10.1186/s12711-022-00724-8

**Published:** 2022-05-13

**Authors:** Anna Wolc, Jack C. M. Dekkers

**Affiliations:** 1 Department of Animal Science, Iowa State University, 806 Stange Road, 239 Kildee Hall, Ames, IA 50010 USA; 2 Hy-Line International, 2583 240th Street, Dallas Center, IA 50063 USA

## Abstract

**Background:**

Bayesian genomic prediction methods were developed to simultaneously fit all genotyped markers to a set of available phenotypes for prediction of breeding values for quantitative traits, allowing for differences in the genetic architecture (distribution of marker effects) of traits. These methods also provide a flexible and reliable framework for genome-wide association (GWA) studies. The objective here was to review developments in Bayesian hierarchical and variable selection models for GWA analyses.

**Results:**

By fitting all genotyped markers simultaneously, Bayesian GWA methods implicitly account for population structure and the multiple-testing problem of classical single-marker GWA. Implemented using Markov chain Monte Carlo methods, Bayesian GWA methods allow for control of error rates using probabilities obtained from posterior distributions. Power of GWA studies using Bayesian methods can be enhanced by using informative priors based on previous association studies, gene expression analyses, or functional annotation information. Applied to multiple traits, Bayesian GWA analyses can give insight into pleiotropic effects by multi-trait, structural equation, or graphical models. Bayesian methods can also be used to combine genomic, transcriptomic, proteomic, and other -omics data to infer causal genotype to phenotype relationships and to suggest external interventions that can improve performance.

**Conclusions:**

Bayesian hierarchical and variable selection methods provide a unified and powerful framework for genomic prediction, GWA, integration of prior information, and integration of information from other -omics platforms to identify causal mutations for complex quantitative traits.

## Background

The goal of genome-wide association (GWA) studies of quantitative traits is to identify genomic regions that explain a substantial proportion of the genetic variation for the trait, with the ultimate goal to identify causal mutations underlying the genetic basis of the trait. The standard GWA approach is to genotype a population that has been phenotyped for the trait(s) of interest and genotyped for many genetic markers across the genome and to analyze these data by estimating and testing the effects of marker genotypes on phenotypes using a regression-type of analysis for each SNP, one at a time [1, 2]. The boom in genotyping technologies, which has increased the number of genomic locations that can be interrogated per individual from several tens or hundreds of restriction fragment length polymorphisms or microsatellites to tens of thousands or millions of single nucleotide polymorphisms (SNPs), has however created several challenges for classical GWA. These include the p >  > n problem (many more marker effects to be tested than available phenotypic observations) and false positives due to population structure. To overcome the latter, the standard GWA approach has been to fit the genotype at each SNP one at a time in a model of phenotype that also fits the effect of population structure, either as principal components of all genotypes across the genome, or by including a polygenic effect with pedigree-based or genomic relationships, or by a combination of these [3, 4]. However, such single-SNP GWA approaches only detect quantitative trait loci (QTL) for which at least one SNP is in substantial linkage disequilibrium (LD) with the causal SNP across the analyzed population. In addition, with limited power, the effects of significant markers tend to be overestimated, the so-called Beavis effect or “winner’s curse” [5, 6]. With large numbers of SNPs tested (most frequently tens of thousands to millions), control of hypothesis testing error rates is important to avoid an excessive number of false positives. Classical multiple-testing corrections such as Bonferroni do not account for dependencies of genotypes at SNPs that are in LD with each other, leading to very high stringency and low experimental power. Instead, Fernando et al. [7] proposed to control the proportion of false positives (PFP) among the positive results, which is independent of the number of tests or correlations among them. This approach is similar to the false discovery rate (FDR) approach that has been applied in many other multiple-testing situations [8].

Some of these issues can be overcome by using the Bayesian multiple regression methodology that was originally developed for genomic prediction [9]. Bayesian methods enable the effects of all genotyped markers to be fitted simultaneously, accommodating different prior distributions of marker effects, variable selection to identify important markers, and using additional sources of information such as other omics data or annotation information [10, 11]. Against this background, the purpose of this paper is to review selected developments in Bayesian models for GWA, for improving the use of information of sequence data, and for using additional omics data to explore biological processes that underlie genetic variation in quantitative traits.

### The Bayesian Alphabet for genomic prediction and GWA

Bayesian multiple regression methods for analysis of quantitative traits using genetic markers were originally developed by Meuwissen et al. [9] for genomic prediction based on availability of high-density marker genotypes, allowing the effects of all genotyped markers to be accounted for simultaneously. These methods included best linear unbiased prediction (BLUP) (later called Bayes-C0 by Kizilkaya et al. [12]), in which a single normal prior is used for the distribution of marker (haplotype) effects, Bayes-A, in which each marker (haplotype) has a normal prior with its own variance, and the Bayesian variable selection (BVS) model Bayes-B, equivalent to Bayes-A but with a prespecified prior proportion, π, of genetic markers (haplotypes) having zero effects. Methods were implemented using Markov chain Monte Carlo (MCMC) sampling using the corresponding prior distributions for the marker (haplotype) effects. Gianola et al. [13] reviewed alternate Bayesian methods for genomic prediction and introduced the term Bayesian Alphabet for the collection of proposed Bayesian genomic prediction methods. They also showed that the Bayes-A method is equivalent to using a single t-distribution as the prior for marker effects. The Fernando group subsequently substantially extended the Bayesian Alphabet by contributing the Bayes-C BVS method [12], in which non-zero effects of markers are sampled from a single normal distribution, with Bayes-C0 as a special case, in which all SNPs are assumed to have non-zero effects and which is equivalent to genomic (G)BLUP. They also introduced the Bayes-Cπ and Bayes-Bπ methods [14], in which the proportion of markers with zero effects is not pre-specified but estimated from the data.

After their introduction for genomic prediction, the Bayesian Alphabet methods, in particular BVS methods such as Bayes-B, were very quickly adapted for use in GWA to differentiate SNPs or windows of SNPs that are associated with phenotype from those that capture just pedigree relationships or noise. Fernando and Garrick [11] provided one of the earlier reviews of the application of the Bayesian Alphabet methods to GWA. In contrast to the single-SNP GWA approaches, Bayesian GWA methods consider the effects of all SNPs simultaneously based on the specified prior distribution of marker effects. However, this also implies that the signal from a causative locus can be captured jointly by a group of SNPs that are in LD with the causal locus, either individually or in combination, i.e. as a diplotype. This contrasts with the single-SNP GWA methods, in which the effect of only one SNP is interrogated at a time. Sahana et al. [15] showed that, if genotypes for the causal loci are not included in the genotype data, which is typically the case when SNP panels are used for genotyping, a window of SNPs around the causative locus can better capture association signals than an individual SNP. The BVS GWA methods were shown to accurately identify association signals in simulated data [16] and have subsequently been used in GWA for many traits in livestock species. In dairy cattle, Kemper et al. [17] showed that the Bayesian multiple regression method called Bayes-R [18], which fits a mixture of normal distributions as prior for marker effects, maps QTL more precisely than the standard single-SNP GWA. Similar results were obtained by Chen et al. [19], who found that Bayesian multiple regression methods resulted in higher accuracy (based on area under the receiver operator curve) for QTL detection in simulated data than single-SNP GWA.

Methods for genomic prediction and GWA using Bayesian Alphabet methods were implemented in the software Gensel by Fernando and Garrick [20], as well as in the BGLR package in R language [21], among others. Computational efficiency of the Bayes-B method for GWA using MCMC was improved by the Fernando group in Cheng et al. [22], resulting in improved implementation of several Bayesian Alphabet methods for GWA in the software JWAS [23]. Moser et al. [10] developed software that implements the BayesR method and popularized it in human GWA applications.

A concern of implementations of the Bayesian Alphabet methods for both genomic prediction and GWA is the computational demand of the MCMC methods that are employed. To overcome this, a fast non-MCMC approach for genomic prediction using Bayes-B based on the expectation maximization (EM) algorithm was developed by Meuwissen et al. [24]. This method also provides estimates of SNP effects, akin to those obtained by Bayes-B. In related work, Stranden and Garrick [25] showed that estimates of SNP effects can be obtained using the standard animal model GBLUP method for genomic prediction by back-solving from genomic estimated breeding values (GEBV), and that the resulting estimates of SNP effects were equivalent to those obtained with the Bayes-C0 approach. This allows GWA based on such a prior to be implemented using standard animal model GBLUP methodology and software, i.e. with the pedigree-based relationship matrix replaced by a genomic relationship matrix. The Fernando group subsequently extended the animal model GBLUP approach for genomic prediction and GWA by weighting the SNPs based on the estimates of their effect when constructing the genomic relationship matrix, as described in Sun et al. [26]. This so-called fast-Bayes-A approach was implemented in an iterative manner using the expectation–maximization (EM) algorithm [26]. Fast-Bayes-A results in estimates that are the maximum likelihood equivalent of the Bayes-A approach, i.e. providing estimates of the mode rather than the mean of the posterior distribution, but using a t distribution as the prior for SNP effects, as in Bayes-A [13]. Further adaptations of the EM approach to accommodate different prior distributions similar to the Bayesian Alphabet were developed by Wang et al. [27] and Chen and Tempelman [28]. Wang et al. [29] developed an efficient Bayes-R approach that is based on a combination of the EM algorithm and MCMC.

Gianola [30] pointed out that a concern with the use of Bayesian Alphabet methods for GWA is that results may heavily depend on the prior(s) used. Accordingly, based on analysis of body weight of broiler chickens, Wang et al. [31] concluded that, compared to the GBLUP method, which assumes a normal distribution as the prior for SNP effects, the Bayes-B method overly shrinks the effects of most genomic regions to zero and, thereby, overestimates the effects of other regions. However, Wolc et al. [32] found that the Bayes-B method was better able to detect and quantify the effects of large QTL for egg weight in layer chickens than GBLUP. They also showed that the accuracy of genomic predictions for egg weight based on Bayes-B was higher and more persistent over generations than the accuracy based on GBLUP, indicating that the estimates of SNP effects obtained with Bayes-B were more accurate. In the end, which prior is most appropriate for GWA likely depends on the genetic architecture of the trait, i.e. on how well the prior fits the real distribution of SNP effects. Because information on genetic architecture is typically limited, it is prudent to implement GWA using different priors for each trait analyzed and compare the results or, alternatively, to fit mixture models such as Bayes-R, which allow proportions for a mixture of prior normal distributions for SNP effects to be estimated from the data [10].

An important advantage of Bayesian multiple regression methods for GWA is that they implicitly account for population structure by fitting all markers simultaneously. Similarly, in single-SNP GWA methods, fitting a polygenic effect based on genomic relationships has been shown to account for population structure and to avoid false positives [33]. Kärkkäinen and Sillanpää [34] found that fitting an additional polygenic effect had limited impact on performance of the Bayesian LASSO method they used, indicating that the simultaneous fitting of all markers adequately accounts for population structure in Bayesian multiple regression methods. For admixed or multibreed populations, fitting admixture proportions or breed composition is typically advocated in order to further reduce false positives from population structure [3]. A review of methods to explicitly account for admixture or breed composition in GWA is in Toosi et al. [35]. However, they argued that, rather than explicitly removing the effect of admixture or breed composition by fitting these population structure effects, GWA should capitalize on the QTL information that is contained in breed differences and showed that this can be accomplished by using BVS methods. Specifically, they found that BVS GWA without explicitly fitting breed composition resulted in higher power to detect QTL than single-SNP GWA with breed composition effects, without inflating the false positive rate. This does assume that breed differences in phenotype are entirely genetic, i.e. due to differences in QTL frequencies, and not in part the result of confounding with environmental factors.

Availability of sequence data is the ultimate p >  > n situation, where all possible genomic locations are interrogated on a usually relatively small number of sequenced individuals, although this can be increased by imputing SNP-genotyped individuals up to sequence if an appropriate sequenced reference population is available. Depending on the LD structure in the population, sequence data, however, does not necessarily improve mapping accuracy, especially when GWA is based on single-SNP methods or GBLUP [36]. There is, however, evidence from human GWA studies, which include larger numbers of unrelated samples than available for most livestock populations, that multi-marker methods that allow variable selection or differential weighting of SNPs result in enrichment of causal variants among the top results [2].

### Using data on both genotyped and ungenotyped animals for GWA

While increasing numbers of animals are being genotyped in breeding programs for different livestock species, still many animals that contribute phenotypic data are not genotyped. For genomic prediction, information from ungenotyped animals was initially incorporated using two-step methods that combined EBV derived using genomic data with EBV derived using pedigree-based BLUP by selection index methods [37]. Others have shown how data from ungenotyped relatives can be incorporated in genomic prediction or GWA as pseudo-phenotypes on genotyped animals, e.g. as pedigree-based daughter yield deviations [38], as deregressed pedigree-based EBV [39], or as family means [40], with appropriate weights on residual terms to accommodate the accuracy of each pseudo-phenotype. Misztal et al. [41] showed how phenotypes from non-genotyped individuals can be incorporated based on pedigree relationships in a so-called single-step GBLUP (ssGBLUP) method and that this increased the accuracy of genomic predictions. Computational methods for ssGBLUP were subsequently advanced by a more direct method to obtain a combined genomic and pedigree-based relationship matrix (the so-called **H** matrix) by Legarra et al. [42] and Christensen and Lund [43].

Wang et al. [27] used the ssGBLUP framework for GWA using different iterative SNP weighting methods to compute genomic relationships, as originally proposed by Sun et al. [26] for GBLUP. Using simulated data, Wang et al. [27] showed that incorporating phenotypes of ungenotyped animals not only improved the accuracy of genomic predictions but also the ability to detect QTL, compared to GBLUP based only on genotyped animals. Subsequently, weighting procedures for ssGBLUP were further optimized by Zhang et al. [44], who found that iteratively deriving weights for windows of neighboring SNPs, rather than separate weights for each SNP, resulted in clearer QTL signals, similar to results obtained with the BVS method when analyzing only genotyped animals.

Fernando et al. [45, 46] extended the Bayesian Alphabet methods to integrate phenotypes on ungenotyped animals for both genomic prediction and GWA. In their approach, genotypes of ungenotyped individuals are imputed using pedigree-based regression methods, while imputation errors are modeled directly to account for uncertainty [47]. These methods have been implemented in the software JWAS [23] to enable single-step Bayesian genomic prediction and GWA using complex models that maximize the use of genomic, pedigree, and phenotypic information. In an application to real data, the implementation of single-step Bayes-B in JWAS allowed identification of more genomic regions associated with infectious hematopoietic necrosis virus resistance in trout than the Bayes-B method that used only data on genotyped animals [48]. Single-step Bayes-B detected similar numbers of QTL as the weighted ssGBLUP GWA method of Wang et al. [27], but less than one third of the detected QTL overlapped between the two methods, emphasizing the potential impact of priors on the results.

### Declaring evidence of QTL for Bayesian multiple-regression GWA

In single-SNP mixed linear model GWA approaches, with each SNP fitted as a fixed effect one at a time, along with a polygenic effect based on a pedigree-based or genomic relationship matrix, significance testing is typically conducted using a standard test for a fixed effect in mixed linear models based on its estimate divided by its standard error. However, Gianola et al. [49] pointed out that fitting a SNP as both a fixed and as a random effect (as part of the genomic relationship matrix) results in a very complex covariance structure for the estimates, with implications for significance testing in the single-SNP GWA, unless the SNP that is treated as a fixed effect is in complete linkage equilibrium with all other SNPs that are treated as random. Duarte et al. [50] showed that the single-SNP GWA test statistic from a linear mixed model that fits the genomic relationship matrix to account for population structure is effectively equivalent to that obtained from back-solved estimates from GBLUP, following [25], divided by the square root of its prediction error variance, which can be obtained from the inverse of the mixed model equations. This allows a GWA to be conducted based on a single GBLUP analysis, rather than a separate analysis for each SNP, similar to obtaining all GWA results from a single run when using Bayesian multiple regression methods. Lu et al. [51] and Aguilar et al. [52] showed that the same holds for ssGBLUP. However, this does not circumvent the multiple-testing problem that is associated with single-SNP GWA.

For Bayesian multiple regression GWA methods, several approaches have been developed to identify which SNPs or windows of SNPs can be considered as explaining a substantial or significant proportion of the genetic variance. Because all SNPs are fitted simultaneously in these methods, these approaches must consider groups or windows of SNPs rather than individual SNPs, because the effect of a causative mutation may be distributed across multiple SNPs. Initial studies used the proportion of variance in GEBV among individuals in the population that is explained by a window or group of SNPs, i.e. the variance across individuals of the window GEBV (computed for each individual and window as the sum across SNPs in the window of the product of the posterior mean and the genotype at each SNP) divided by the variance of genome-wide GEBV of those same individuals [53]. Onteru et al. [54] and Fan et al. [55] derived significance thresholds for these proportions using bootstrap methods. However, this requires a GWA for every bootstrap sample, which is a computationally very demanding. As a result, this approach has not been used extensively.

An alternative criterion to identify association signals in Bayesian multiple regression GWA is an estimate of the proportion of genetic variance that is explained by a group or window of SNPs. Instead of a ratio of variances of GEBV, as in Boddicker et al. [54], this criterion is a ratio of estimates of the variance of true breeding values for the window and the variance of genome-wide true breeding values. This criterion was first implemented by Wolc et al. [40] by using the concept that after burn-in, the SNP effects at a given iteration of the MCMC are sampled from the posterior distribution of SNP effects. Thus, the (window) breeding values that can be computed for each individual based on the sampled SNP effects are draws from the posterior distribution of those breeding values, as are the variances of those breeding values across individuals. In contrast to the approach by Onteru et al. [54], this approach does not require bootstrapping but is implemented as part of the MCMC for GWA of the original data. These concepts were implemented in Gensel 4.0 [56], providing posterior distributions and means of the variance of breeding values for non-overlapping 1-Mb windows across the genome, as well as for the genome-wide breeding values and the ratio of these variances for each iteration of the MCMC. However, de los Campos et al. [57] pointed out several issues with inferences about genetic variance from multiple marker regression models when the causal loci are not genotyped, including misspecification of the likelihood function, and lack of consistency and bias of estimates.

The posterior distribution of genetic variance explained by a window from the approach described in [40] also enables calculation of the posterior probability that a window or group of SNPs explains non-zero genetic variance or more genetic variance than expected under a polygenic model, i.e. with a uniform distribution of genetic variance across the genome. Whether the use of these posterior probabilities results in proper control of false positives and negatives under specific frequentist-based hypotheses related to the genetic variance that is explained by a window or by group of SNPs has to our knowledge not been investigated.

For BVS methods, the MCMC samples after burn-in can also be used to compute the proportion of samples for which a particular SNP was included in the model with a non-zero effect, which is referred to as the posterior probability of association (PPA) for that SNP. Similarly, the proportion of samples for which at least one SNP in a window was included in the model can be computed, which was referred to as the window posterior probability of association (WPPA) by Fernando et al. [58]. The WPPA was initially introduced as a significance criterion by Sahana et al. [15]. Using simulation, Fernando et al. [58] showed the value of WPPA in the identification of association signals with control of the posterior type I error rate. They also showed how Bayesian multiple regression methods reduce the problem of signal dependence, which refers to SNPs that are some distance from QTL and exhibit a GWA signal, and was identified as an issue for linkage analysis by Chen and Storey [59]. Fernando et al. [58], however, showed that simultaneously fitting all SNPs in the region results in a concentration of the association signals to a small region around the QTL, especially when based on LD. In a recent simulation study by Lima et al. [60], WPPA was found to be superior to other methods in terms of power to detect QTL for both traits with oligogenic and polygenic architectures. However, in follow-up work by the Fernando group, Li et al. [61] observed that WPPA may lead to spurious associations when the distribution of SNPs across the genome is uneven. To address this issue, they proposed two easy-to-implement methods, with good results, i.e., dividing the genome into windows with a fixed number of SNPs, or adjusting the WPPA for SNP density. In related work, Legarra et al. [62] developed a method using Bayes factors to evaluate genomic windows but did not fully justify a significance threshold for this method.

### Bayesian GWA models to detect pleiotropic QTL

In addition to analysis of individual traits, there is also an interest in understanding genetic correlations between traits, with particular interest in genomic regions that break unfavorable genetic correlations. Many studies have attempted to identify pleiotropic QTL regions by comparing results from single-trait GWA, i.e. by identifying overlap between windows that explain a large proportion of genetic variance across traits [63]. This is, however, hampered by the typically limited power of GWA, which leads to many false negatives and, as a result, limited overlap between significant windows for two traits that may in fact have a high genetic correlation. In a more direct approach, Gorbach [64] used correlations and covariances of window GEBV of individuals from univariate genomic prediction analyses of two traits to identify pleiotropic regions. Applied to growth rate and feed intake in pigs, they identified several regions for which the correlation between window GEBV for these two traits was opposite to the expected undesirable positive genetic correlation between these two traits (i.e. cryptic pleiotropic regions). Using a similar approach, Bolormaa et al. [65] used the covariance between window GEBV for two traits divided by the product of the phenotypic standard deviation for each trait. Such a criterion is preferred over the correlation between window GEBV used by Gorbach [64] because the latter could be high for windows that explain very little genetic variance for one or both traits and which, therefore, contribute little to the genome-wide genetic correlation between the traits. In general, a problem with these window GEBV approaches is that predictions of breeding values are affected by both genetic and random environmental effects (they are a linear function of phenotypes) and, therefore, their correlations and covariances are not proper estimates of genetic correlations or covariances. In a second approach, Bolormaa et al. [65] computed the probability that a SNP had no association with any trait as the product of (1-PPA) for that SNP from each of the single-trait BVS analyses. Note that this approach can be extended to identify pleiotropic genomic windows by using WPPA instead of PPA. The two approaches used by Bolormaa et al. [65] to detect pleotropic SNPs or regions, i.e. based on the covariance of window GEBV and based on the product of (1-PPA), detected similar pleiotropic genomic regions and SNPs but these regions were different from another approach used by Bolormaa et al. [66] based on multi-trait analysis of single trait results from single-SNP GWA. These comparisons were, however, based on somewhat arbitrary significance thresholds that may not control the same error rates.

In a more formal approach, Jia and Jannink [67] extended the single-trait Bayesian multiple regression models to multi-trait analyses by specifying a multi-variate prior distribution for marker effects. Although the focus of their study was to increase the accuracy of genomic prediction, these same models can also be used to identify and estimate the effects of pleiotropic QTL, especially when using BVS methods. However, when using BVS, Jia and Jannink [67] limited variable selection by allowing SNPs to only have a non-zero effect on either all or none of the traits. A similar approach was used by Calus and Veerkamp [68]. A more flexible model, in which any SNP can have an effect on any combination of traits, was used by Cheng et al. [69] and has been implemented in the JWAS software [23].

In the multi-trait Bayesian GWA approaches of [67–69], the multi-variate prior distributions used for SNP effects assume the same correlation for all SNPs. To avoid the potential impact of such priors on GWA results, Kemper et al. [70] developed and implemented a multiple trait Bayesian multiple regression method called Bayes-MV, with a mixture of prior distributions for SNP effects following Bayes-R. These distributions were assumed to be independent between traits but with a specified prior proportion of SNPs having no effect on any trait. Significance of effects was based on the mean posterior probability that a SNP had a non-zero effect on any trait, but this criterion can be expanded to windows of SNPs, as in Fernando et al. [58]. Using simulated data, Kemper et al. [70] showed that the Bayes-MV method detected a larger number of true QTL than the equivalent single-trait method that assumed no SNPs with zero effects, i.e. Bayes-R.

In summary, Bayesian multiple regression methods provide a flexible approach for the detection of pleiotropic QTL. However, the impact of prior assumptions on SNP effects across traits requires further investigation. Also, an important limitation of all methods that attempt to use genotype–phenotype associations to detect pleiotropic QTL is that they cannot differentiate the presence of a pleiotropic QTL from the presence of two closely linked single-trait QTL, depending on the extent of LD in the region. In addition, similar to the issues with inferences about genetic variance based on multiple-marker regression models raised by de los Campos et al. [57], estimates of genetic covariances and correlations based on multiple-marker regression models can misrepresent the true genetic parameters if the causal loci are not genotyped because of incomplete LD between markers and QTL and among QTL, as demonstrated by Gianola et al. [71].

Identification of pleiotropic QTL is important for understanding the biology behind traits and multi-trait GWA approaches are expected to increase power to detect QTL and increase the accuracy of genomic prediction. However, it is not clear whether pleiotropic QTL should receive specific attention in multi-trait breeding programs, beyond the emphasis they receive in standard total merit selection criteria based on GEBV.

### GWA using Bayesian structural equations models

Structural equation models (SEM) aim at going beyond correlations to making inferences on causal relationships between variables, as first proposed by Wright [72] based on path analysis. Adaptations of SEM to quantitative genetics and mixed models were proposed by Gianola and Sorensen [73], including methods to infer causal relationships among traits. A review of applications of SEM in the context of analysis of relationships between traits in animal breeding is in Inoue [74]. Valente et al. [75] clarified that, although SEM allow causal relationships between phenotypic traits to be investigated, they offer no advantage over multiple-trait models for standard multiple-trait selection purposes. This is because genetic correlations are (primarily) caused by pleiotropic QTL and pleiotropy has no causal direction, i.e. a QTL can be pleiotropic for two traits by either affecting both traits directly or by affecting one trait directly and the other indirectly via the causal influence of the first (assuming that both traits are causally connected). Thus, selection on trait 1 is expected to result in a correlated response in trait 2 by changing allele frequencies at pleiotropic QTL, regardless of its causal relationships with phenotypes for the two traits. However, Valente et al. [75] described a number of scenarios in which the additional information that is obtained from SEM can be beneficial, including allowing more accurate estimation of breeding values under new (unobserved) environmental conditions in the case of genotype-by-environment interactions.

Li et al. [76] applied SEM to QTL mapping by differentiating between direct and indirect effects of a QTL on a trait. They distinguished three groups of QTL for a two-trait scenario based on their direct effects: (i) QTL that directly affect only trait 1; (ii) QTL that directly affect only trait 2; and (iii) QTL that directly affect both traits. Note that QTL that directly affect only trait 1 (2) can have indirect effects on trait 2 (1) through a causal effect of the phenotype for trait 1 (2) on trait 2 (1). In addition, QTL that directly affect both traits can have two paths of effects on a trait, i.e. direct effects and indirect effects through the other trait. Thus, in contrast to multiple-trait models, which model the overall effects (direct plus indirect) of QTL or markers, SEM differentiate between the direct and indirect effects of QTL or markers.

Momen et al. [77] adapted the SEM-QTL model to single-SNP SEM-GWA for quantitative traits based on a mixed linear model with fixed effects for a single SNP (i.e. similar to a single-SNP GWA for individual traits), with application to the estimation of the direct and indirect effects of SNPs on traits in broiler chickens. Wang et al. [78] implemented multi-marker SEM-GWA BVS models, introducing the SEM Bayesian Alphabet. They showed that SEM-GWA BVS models have similar or greater power to detect QTL than multi-trait BVS, but provide greater insight into biological mechanisms of the effects of QTL on traits through direct and indirect effects. These methods were recently applied to the analysis of the structural genomic relationships and QTL for milk proteins in dairy cattle by Pegolo et al. [79]. Bayesian multi-SNP SEM-GWA has also been implemented in human genetics using Bayesian graphical models by Briollais et al. [80] and for meta-analysis using Bayesian network analysis by Zhang et al. [81].

### Use of functional information for (post-)GWA analysis

Given the typically limited statistical power and mapping precision of GWA studies, interpretation of GWA results requires substantial post-GWA analyses. These can be based on evidence from other -omics data, results from previous QTL or GWA studies, and functional biological information about genes located in the identified genomic regions, as reviewed by Uffelmann et al. [2]. Ramanan et al. [82] proposed that one way to gain additional insight into GWA results and the importance of different biological pathways is to jointly evaluate the evidence of association for groups of markers or genes that are part of the same biological pathway. One strategy is to use enrichment analyses to determine whether the top GWA regions are enriched for genes with certain biological features, such as membership of a biological pathway or having certain gene ontology (GO) terms. This requires setting a significance threshold for regions to include in the enrichment analysis, which can have a large impact on results. An alternative is to use all GWA results in a ranked gene set enrichment analysis, as proposed by Subramanian et al. [83] and implemented in their GSEA software. This requires GWA results to be assigned to each gene in the genome. For single-SNP GWA, this could be based on the average − log(p-value) of all SNPs in or around a gene. For Bayesian multiple regression GWA, a possible rank criterion is the percent of genetic variance explained by each genomic window. This, however, would result in the same value to be assigned to all genes located in a window, which causes problems in the analyses. An alternative that was suggested by Delpuech [84] and employed by Cheng et al. [85] is to assign biological features to windows based on the features of genes that are present in the window.

Rather than for post-GWA analyses, functional biological information can also be integrated in the GWA analysis. One strategy is the feature GBLUP approach employed by, e.g., Edwards et al. [86]. In this approach, SNPs are classified based on genomic features and a separate random genetic effect is fitted for each class of SNPs in a mixed linear GBLUP model, using a genomic relationship matrix derived using genotypes for that class of SNPs. Estimation of the variance component for each feature class then allows different weights to be assigned to different classes of SNPs. When working with SNP genotype or sequence data, feature classes can be assigned based on genome annotation, candidate gene lists, or known causal variants [87].

An alternative approach is to incorporate prior biological information in the Bayes-R approach of Kemper et al. [17], as proposed by MacLeod et al. [87]. In their Bayes-RC method, the mixture of normal prior distributions with different variances of Bayes-R was cross-classified with feature classifications of the analyzed SNPs or variants. This approach allows the model to estimate the mixture of each of the variance size distributions of Bayes-R for each feature class. The Bayes-RC method was shown to outperform Bayes-R in terms of power and precision of QTL discovery in simulated and real dairy cattle milk production phenotype data [87]. Xiang et al. [88] used the Bayes-RC method to successfully prioritize potential causative variants for multiple traits across populations using (imputed) whole-genome sequence data on large numbers of dairy cattle, combined with classification of sequence variants based on functional information.

### Combining multi-omics data for prediction and fine-mapping using Bayesian multiple regression methods

Combining multiple omics data to achieve improved phenotype prediction and fine-mapping of causal loci can be performed in a stepwise post-GWA interpretation of GWA results, using other -omics data, meta-data from other GWA, and functional information, as reviewed by Uffelmann et al. [2]. Alternatively, other -omics data can be directly integrated into the GWA. Huang et al. [89] provide a recent review of methods to integrate multi-omics data for phenotype prediction in the context of precision medicine. In this context, Bayesian methods can provide a very flexible structure that can be used to extract information from multi-omics data individually or simultaneously. For example, Wang et al. [90] developed a hierarchical BVS model to integrate information from different -omics platforms to identify genes or biomarkers that are associated with a phenotypic outcome. Fang et al. [91] extended this method to allow for large amounts of missing data, since the different sets of -omics data are typically available on substantially non-overlapping groups of individuals. Xiang et al. [92] used information on functional annotation and evolutionary conservation to improve mapping precision for production traits in cattle. Maity et al. [93] used a Bayesian SEM to integrate information from copy number variants and gene expression to predict survival of cancer patients by specifying and predicting a number of latent variables that underlie the copy number and gene expression data. Bayesian prioritization models can be used to narrow down hits from transcriptome-wide association (TWA) studies to gene sets that include causal loci with a predefined probability [94] and use information of both cis and trans acting expression QTL (eQTL) [95]. Bayesian sparse linear mixed models (BSLMM) [96] fit all SNPs nearby a gene into a model with two distributions, allowing larger and polygenic effects on gene expression. Based on a BSLMM, Wheeler et al. [97] found *cis* gene expression regulation to be mostly oligogenic.

An additional level of information that can inform causal relationships between genotype and phenotype is the proteome [98], which in addition to interpretation of GWA and TWA results, can provide targets for future functional studies and drug targets [99]. Proteome-wide association studies aggregate the signal of all variants that jointly affect a protein-coding gene and assess their overall impact on the protein’s function and the phenotype of interest [100].

Flutre et al. [101] introduced an across-tissue Bayesian model that allows sharing of a proportion of eQTL across tissues, accounting for correlations among tissues within individuals. Across-tissue analysis helps to partly overcome the issue of limitations in the availability of expression data for different tissues, which affects most studies and is due to the still relatively high cost of generating genome-wide gene expression data. In a similar manner, but using proteomic data, an algorithm called LOCUS [102] borrows strength across correlated protein levels and DNA markers on a genome-wide scale to effectively increase statistical power.

Another solution to missing data is to impute missing records using integrative techniques that use correlations and shared information across data sets [103, 104]. The integrative risk gene selector (iRIGS) [105] is a Bayesian framework that integrates multi-omics data and gene networks to infer risk genes for identified GWA signals. Bayesian tests of colocalization [106] integrate GWA results with eQTL, methylomics, or other -omics data to provide insights into context specific gene regulation [107]. Associations between protein levels and variation in DNA sequence that colocalize with disease risk alleles can suggest disease-associated pathways, revealing novel drug targets and translational biomarkers.

Hajiramezanali et al. [108] proposed a graph-structured data integration method called Bayesian Relational Learning (BayReL) for integrative analysis of multi-omics data and applied it to explore microbiome-metabolome interactions in cystic fibrosis. They also applied the method to identify miRNA-mRNA interactions in breast cancer by integrating gene expression profiles and in vitro sensitivity of tumor samples to chemotherapy drugs. Zhu et al. [109] proposed another type of directed algorithm called MRLocus to investigate how perturbations of gene expression or individual regulatory elements affect downstream phenotypes.

### Challenges

While BVS methods continue to be improved, they remain computationally demanding, which in the era of increasing availability of sequence data on tens or hundreds of thousands individuals with millions of SNPs becomes a challenge. Improved Gibbs-samplers and non-MCMC algorithms are expected to alleviate some of the computational burden. van der Berg et al. [110] proposed to reduce computational demands of BVS methods, while maintaining the accuracy of marker effect estimation, by splitting the sequence data by chromosome and dropping variants with small effects. Another strategy to reduce the number of variants is to use single-SNP GWA to preselect variants or to inform priors, but ideally this should be done on a separate data set, which in most cases is not available. Another possibility is analysis with LD pruned data, followed by full marker saturation only in regions with evidence of association. Strong LD can also limit the ability to identify causal mutations even if all markers can be fitted simultaneously. Today this is mostly addressed by gathering large sample sizes with the hope of finding enough individuals with cross overs to break the LD in order to narrow candidate regions. Increasing understanding of gene expression, regulation, and interaction will help to develop better priors for Bayesian multiple regression GWA methods, which can help narrow candidate regions. Ultimately, functional genomics and gene editing approaches are required to validate putative causal QTL.

## Conclusions

In addition to genomic prediction, Bayesian multiple marker regression methods provide a flexible and reliable framework for GWA studies and for integrating multiple sources of functional and genomic information (multi-omics data) to gain insight into the genetic architecture of complex traits. Further development of methods that are less dependent on choice of priors or that include more appropriate priors is, however, warranted, as well as of methods for integration of multi-omics, functional, and biological information.

## Data Availability

Not applicable.
